# Medical Expert Evidence.—I

**Published:** 1909-04-24

**Authors:** 


### Medico-Legal Points.

### MEDICAL EXPERT EVIDENCE.—I.

Medical men, when summoned before courts of law, may appear as ordinary witnesses, to state facts within their own knowledge, or as skilled witnesses to interpret them. In the former position their testimony differs in nothing from that of other persons, and is subject to the same rules of admissibility and interpretation. Their profession imparts no additional value to their testimony in such cases, for it is clear that they simply testify to those matters to which any equally intelligent layman might testify. In these cases their testimony is restricted to matters of personal knowledge alone, and their opinions become inadmissible. It is well to understand this at the outset, and the medical man, before going into the witness-box, should make certain in what capacity he is called. Both capacities are to a certain extent often united in him, but as a general rule it is not usual to call an expert to prove what an ordinary witness can, nor would he be allowed to if no reason of a scientific character existed to justify it.

But, on the other hand, when he takes his place as an expert before a court, a legal paradox is instituted in his behalf, by which he is allowed to testify not to what he knows, but to what he believes or forms an opinion upon, based necessarily on probabilities of analogy as well as experience. His testimony becomes a generalisation of facts, by means of which he undertakes to explain certain phenomena, or particular instances, as deductions from a law of common authority and government over such facts. He first generalises, and then abstracts, and his opinion expresses the degree of agreement between a general law and the particular subject of its authority.

In view of the immense erudition required to make a skilled witness in medicine, it follows that the high and responsible position occupied by the medical expert before courts of justice renders it indispensably necessary for him to possess the greatest measure of proficiency in those matters about which he is called to testify. And though he need not be a schoolman, nor skilled like a lawyer in dialectics and rhetorical fencing, he should at least be able to distinguish between the real and the apparent in those physical phenomena specially appertaining to that human nature of which he is the accredited minister and interpreter. Much of that wrangling over the taking of skilled testimony before courts, which constitutes the opprobrium of so many trials, may be attributed to a want of precision and candour in putting questions, and the consequent inability of witnesses to answer them clearly. There being always an *arrière pensée* to every question put by counsel, while the expert is confined to direct answers, his manner, whether brief and outspoken or halting and ambiguous, is ever liable to be misintepreted, according as it approximates to or departs from the point intended to be reached by his examiner.

There is on this account among medical men a great sensitiveness and reluctance about appearing before courts, for, besides being at times roughly handled in a cross-examination upon professional subjects by those of a different calling, their opinions are often disregarded by the jury, who pronounce a verdict directly antagonistic to the current of their testimony. To be summoned to express an opinion in a case, and yet not have that opinion form part of the purport of the judgment, seems to many little short of an insult. But this view involves an entire misapprehension of the duties and scope of testimony of an expert. He should understand at the outset that he is not called to express an opinion upon the merits of the case; that he has no proper concern in the issue, and by whichever party called he is in no wise the witness, much less the advocate, of that side. His testimony is invoked, in almost an impersonal sense, to explain the relations of cause and effect in certain physical facts that are in evidence before the court, and which relations, being unintelligible to the jury, require professional explanation at his hands in order that due weight may be given to the facts out of which they arise. His duties are properly limited to gauging the value of certain facts as they appear in evidence—facts whose importance to the issue cannot be determined without his assistance. But an opinion upon the relations of facts is not an opinion upon the truth of those facts. It is for him to decide the former; it is for the jury to decide the latter. For him to pronounce an opinion either upon the truth of the facts given him for interpretation, or upon the merits of the case, would be to usurp the province of the jury, and thus incorporate in his own person the functions of court, advocate, and witness. The witness cannot in strictness be asked his opinion respecting the very point which the jury are to determine. For instance, if the question be whether a particular act for which a prisoner is tried were an act of insanity, a medical man conversant with that disease, who knows nothing of the prisoner, but has simply heard the trial, cannot be broadly asked his opinion as to the state of the prisoner's mind at the time of the alleged crime, because such a question involves the determination of the truth of the facts deposed to, as well as the scientific inference from those facts. Yet he may be asked what judgment he can form on the subject, assuming the facts stated in evidence to be true. It is, therefore, from no desire to diminish either the importance or the value of medical testimony that courts are compelled to adopt such rules. And that they are wisely conceived must be apparent to all who will pause to inquire what would be the effect of allowing experts to usurp power which they might be tempted to use for the benefit solely of the party calling them.

In the examination of experts it often happens that neither counsel nor witness understand one another, each using terms of various signification, according to the import most usually given them by either profession. This necessarily produces ambiguity and confusion, a condition not undesired by him who seeks to make the worse appear the better reason, but always to be reprobated in a court of justice, where truth rather than victory should be sought after. For these reasons the testimony of experts, constituting a most responsible branch of the law of evidence, since it is something higher than a mere oral recitation of facts, being an opinion *ex cathedra* upon the value of such facts, and thus quasi-judicial, is jealously criticised to see that it is tainted by no bias towards either party. Under a distorted phraseology the witness might easily be made to appear prejudiced, or an undue emphasis placed upon a statement of subordinate consequence might elevate it into the sphere of a dogmatic assertion. Interpreting an idea as expressed through language, or construing that language technically and etymologically, instead of in its usual and customary senses, may occasion wide differences of mutual comprehension, so that, in fact, throughout the whole examination of an expert, more even than in the case of an ordinary witness, the doctrines of liberal rather than close construction should be applied.

Opinions in general are not evidence, for they are plain and palpable violations of the germinal principle of all evidence, which is personal knowledge directly acquired. They are, then, either something more or something less. The Roman law, it would seem, speaking of physicians when called as experts, evidently considered them more in the light of a day when juries were unknown, and the practitioner could easily merge his opinion in that of the expert, thus, in fact, converting the witness into the court itself (Maynz, "Elemens de Droit Romain," vol. i. p. 348). Such a substitution as this was never contemplated at common law, where the provinces of judge, juror, and witness are strictly prescribed and most jealously guarded, and where any encroachment on the part of either would invalidate the judgment, or at least render it amenable to revision.

The opinion rendered by the expert witness should involve so much of special knowledge as to exclude it from the sphere of ordinary testimony, since, if it passes within it, the essential prerequisite element of skill is thereby destroyed, and the expert changes into an ordinary witness. And therefore no party is entitled to ask the opinion of a professional witness upon any question except one of skill or science. The more the ground of expert testimony can be narrowed and circumscribed, the easier it will be to obtain from its assistance intelligible and satisfactory results; while, on the other hand, the looser the system exhibited in introducing it before courts and permitting counsel to stretch it *ad infinitum*, the more confusing and useless it becomes, even if, through astute perversion, it does not work harm to both causes of science and justice.

